# Mitochondria as the Target of Hepatotoxicity and Drug-Induced Liver Injury: Molecular Mechanisms and Detection Methods

**DOI:** 10.3390/ijms23063315

**Published:** 2022-03-18

**Authors:** Milos Mihajlovic, Mathieu Vinken

**Affiliations:** Department of Pharmaceutical and Pharmacological Sciences, Vrije Universiteit Brussel, Laarbeeklaan 103, 1090 Brussels, Belgium; milos.mihajlovic@vub.be

**Keywords:** hepatotoxicity, liver injury, mitochondrial dysfunction, molecular mechanisms, in vitro

## Abstract

One of the major mechanisms of drug-induced liver injury includes mitochondrial perturbation and dysfunction. This is not a surprise, given that mitochondria are essential organelles in most cells, which are responsible for energy homeostasis and the regulation of cellular metabolism. Drug-induced mitochondrial dysfunction can be influenced by various factors and conditions, such as genetic predisposition, the presence of metabolic disorders and obesity, viral infections, as well as drugs. Despite the fact that many methods have been developed for studying mitochondrial function, there is still a need for advanced and integrative models and approaches more closely resembling liver physiology, which would take into account predisposing factors. This could reduce the costs of drug development by the early prediction of potential mitochondrial toxicity during pre-clinical tests and, especially, prevent serious complications observed in clinical settings.

## 1. Introduction

Drug toxicity is a ubiquitous clinical problem that can have far-reaching consequences, from the drug development process to the healthcare system, with huge costs and major implications on patient safety, which is reflected by increased morbidity and mortality [1]. The liver is one of the most affected organs in drug toxicity, as seen both during drug development and pre-clinical safety studies, and especially following marketing [1,2,3]. Drug-induced liver injury (DILI), under the most severe circumstances, can lead to the need for liver transplantation and even the patient’s death [4].

Even though there are various mechanisms responsible for DILI, some of which are not yet fully investigated or known, one process that is often present and described for numerous drugs is mitochondrial damage and dysfunction [5,6]. A drug can have that effect directly or indirectly, exerted by its intermediate metabolism products [6,7]. Hepatotoxicity linked to mitochondrial dysfunction can be due to damage to mitochondria and their components, leading to a wide range of consequences and injury types to the liver. Typically, altered energy production, excessive oxidative stress, the release of pro-apoptotic signals triggering cell death, and altered lipid metabolism leading to triglyceride accumulation (steatosis) and steatohepatitis are observed [8]. Given the importance of DILI and drug-induced mitochondrial dysfunction, it is essential that these adverse reactions are detected early on during the drug development process.

In the present review, we first describe briefly the main mitochondrial functions and their relevance for the liver. Next, we review the main mechanisms of liver injury with a particular focus on the process of drug-induced mitochondrial dysfunction. We also discuss some key factors influencing DILI and mitochondria-related hepatotoxicity, as well as the most common experimental tools and methods used to evaluate mitochondrial dysfunction. Finally, we conclude with a discussion regarding future perspectives and the need for novel and integrative approaches for predicting drug-induced mitochondrial injury and hepatotoxicity.

## 2. Mitochondria and the Liver

### 2.1. Mitochondrial Functions

**Figure 1:**
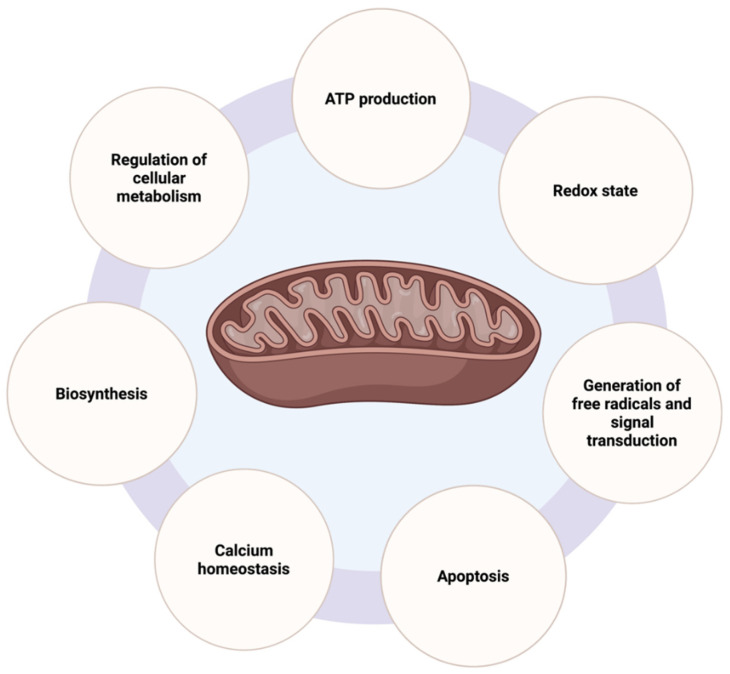
Schematic representation of the main mitochondrial functions in a cell. Created with Biorender.com (accessed on 21 February 2022; Toronto, ON, Canada) (ATP—adenosine triphosphate).

Mitochondria are organelles of bacterial origin, composed of two membranes surrounding the matrix containing enzymes and mitochondrial DNA (mtDNA). They are indispensable for the normal function of eukaryotic cells, as evidenced by their role in energy production, the regulation of cellular metabolism, and apoptosis (Figure 1) [9,10,11,12]. Due to the presence of major complex enzymatic systems and via the tricarboxylic acid (TCA) cycle and the electron transport chain (ETC), mitochondria are the main site of adenosine triphosphate (ATP), and therefore energy production, in healthy cells, starting from the oxidation of sugars, fatty acids, and amino acids [13]. Furthermore, mitochondria participate in the biosynthetic pathways of glucose, amino acids, fatty acids, cholesterol, and heme, but also in calcium homeostasis and the disposal and re-purposing of cellular waste, such as ammonia, hydrogen disulfide (H_2_S), and reactive oxygen species (ROS), through various pathways [11,14]. Mitochondria also regulate programmed cell death by participating in the intrinsic pathway of apoptosis, which requires mitochondrial outer membrane polarization (MOMP), cytochrome c release, and subsequent caspase 3 and 7 activation [12]. Various mitochondrial proteins possess pro-apoptotic or antiapoptotic potential, such as the B cell lymphoma 2 (Bcl-2) family of proteins, and can directly lead to apoptosis upon specific stimuli, including growth factor withdrawal, mitotic arrest, and DNA damage [12]. Overall, proper mitochondrial functioning is of utmost importance and mitochondrial defects or alterations in their activity can evoke various diseases, such as metabolic and neurodegenerative disorders [15].

### 2.2. Liver Metabolic Functions and Mitochondrial Activity in Hepatocytes

**Figure 2:**
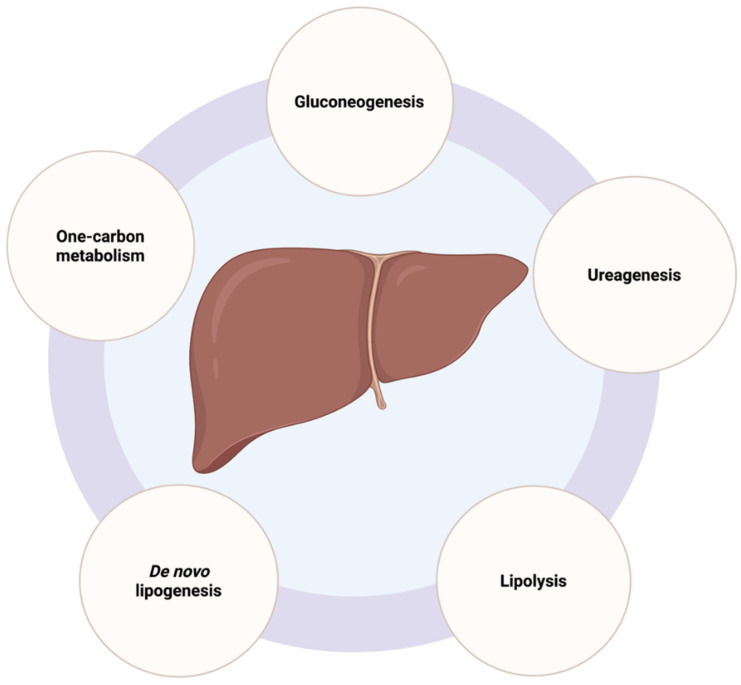
Schematic representation of the main hepatic metabolic functions in which mitochondria have a critical role. Created with Biorender.com (accessed on 21 February 2022; Toronto, ON, Canada).

The liver is the most important organ in the body when it comes to maintaining energy homeostasis, regulating the storage and metabolism of nutrients, and blood detoxification. It is an extremely metabolically active organ and one of the richest organs in terms of the number of mitochondria. Hepatocytes, the most abundant cell type of the liver, are highly specialized in various metabolic activities, both anabolic, such as gluconeogenesis, lipogenesis, and glutaminogenesis, as well as catabolic, including glycolysis, lipolysis, and ureagenesis (Figure 2). Mitochondria play key roles in each of these biochemical events [16].

#### 2.2.1. Gluconeogenesis

Mitochondrial enzymes are responsible for gluconeogenesis upon the prolonged fasting and depletion of glycogen stores starting from precursors, such as lactate, glycerol, and amino acids [17]. Lactate is oxidized to pyruvate by lactate dehydrogenase (LDH), which is transported into mitochondria, where it becomes transformed into oxaloacetate by pyruvate carboxylase. Oxaloacetate is reduced to malate in a reaction catalyzed by malate dehydrogenase and exported into the cytoplasm, where it is converted to phosphoenolpyruvate by cytoplasmic phosphoenolpyruvate carboxykinase [18]. Glucogenic amino acids are first converted to α-ketoacids via deamination reactions and further to precursors of gluconeogenesis, such as pyruvate and oxaloacetate, while glycerol is converted to glycerate-3 phosphate, serving as a precursor of gluconeogenesis. Phosphoenolpyruvate and glycerate-3 phosphate are subsequently metabolized to glucose-6-phosphate and, finally, glucose [17].

#### 2.2.2. De Novo Lipogenesis

In addition to gluconeogenesis, mitochondria are also key players in the synthesis of fatty acids in the process known as de novo lipogenesis. The starting metabolite in this process is acetyl coenzyme A (CoA) formed in the TCA cycle when there is an excess of the main nutrients, glucose, and amino acids as well as alcohol [19]. The excess of synthesized acetyl-CoA is transported to the cytoplasm in the form of citrate, where the synthesis of fatty acids is completed. Citrate is first transformed into acetyl-CoA by ATP-citrate lyase, which in the following two steps is converted first into malonyl-CoA by acetyl-CoA carboxylase and finally into palmitate by fatty acid synthase [17]. In the final step, palmitate is converted to fatty acids by stearoyl-CoA desaturase 1 [17]. In the liver, the expression of acetyl-CoA carboxylase and fatty acid synthase is regulated by glucose and insulin through the transcription factors carbohydrate-response element-binding protein (ChREBP) and sterol regulatory element-binding protein 1c (SREBP-1c) [20].

#### 2.2.3. Urea Cycle

Like for gluconeogenesis and lipogenesis, hepatocytes are equipped with the necessary enzymatic machinery to synthesize urea from ammonia in the urea cycle, which also involves mitochondria [19]. The first steps of the urea cycle take place in mitochondria and include the conversion of ammonia to carbamoyl phosphate catalyzed by carbamoyl phosphate synthase-1 and the subsequent formation of citrulline in a reaction of the condensation of carbamoyl phosphate and ornithine catalyzed by ornithine carbamoyltransferase [21]. Citrulline is then transported into the cytoplasm, where together with aspartate, argininosuccinate is formed, facilitated by argininosuccinate synthase, which is transformed into fumarate and arginine by argininosuccinate lyase, and that finally produces ornithine and urea [21].

#### 2.2.4. Lipolysis

Following lipolysis, in which lipid triglycerides are hydrolyzed into glycerol and fatty acids, the latter are further metabolized in a catabolic process called β-oxidation, which takes place in hepatic mitochondria [17]. This process occurs either directly after lipolysis or following initial peroxisomal β-oxidation that shortens very-long-chain and polyunsaturated fatty acids before they reach the mitochondria [22]. Short-chain and medium-chain fatty acids can freely enter mitochondria, whereas long-chain fatty acids need to be transformed into acyl-carnitine by carnitine palmitoyltransferase 1 (CPT1) and transported into the mitochondrial matrix via acyl-carnitine translocase [19]. Once inside mitochondria in the form of acyl-carnitine intermediates, long-chain fatty acids are transformed back into acyl-CoA by carnitine palmitoyltransferase 2, which separates the acyl group from carnitine, whereas short-chain and medium-chain fatty acids are activated to acyl-CoA molecules by specific acyl-CoA synthases. Acyl-CoA derivatives then enter the β-oxidation cycle, consisting of four reactions of dehydrogenation, hydration, oxidation, and thiolysis, in each of which it is shortened by two carbons with the generation of acetyl-CoA moieties until the complete oxidation of the original acyl-CoA derivative [17,19]. Acetyl-CoA moieties released during β-oxidation are ready to be used either in the TCA cycle or for the synthesis of ketone bodies (acetylacetate, β-hydroxybutyrate, and acetone), which are oxidized for energy purposes in peripheral tissues, including the brain, kidney, and muscle. In this way, hepatic mitochondrial β-oxidation and ketogenesis contribute to energy homeostasis [19,23]. Peroxisome proliferator-activated receptor alpha (PPARα), a nuclear receptor family member activated by long-chain fatty acids and phosphatidylcholines, is a major regulator of fatty acid β-oxidation in mitochondria and peroxisomes [24,25].

#### 2.2.5. One-Carbon Metabolism

Hepatic mitochondria also possess a complete set of enzymes needed for one-carbon (1C) metabolism, which includes both the methionine and folate cycles and that serves to generate methyl groups (one-carbon units) used for biosynthetic processes [26], in particular, the synthesis of purine and thymidine, polyamines amino acids, phospholipids, and creatinine, as well as for the methylation reactions of DNA, RNA, and proteins [27]. Dietary folic acid serves as a universal 1C acceptor. It is converted first to dihydrofolate and then to tetrahydrofolate in the enzymatic reactions catalyzed by dihydrofolate reductase, which then accepts 1C units derived from amino acids, including serine and glycine [28]. This process leads to the formation of methylene-tetrahydrofolate, which donates its 1C unit to thymidylate synthesis [28]. Methylene-tetrahydrofolate can also be converted to methyl-tetrahydrofolate by methylenetetrahydrofolate reductase, which participates in methionine recycling, or it can be converted to methenyl-tetrahydrofolate and subsequently to formyl-tetrahydrofolate by methylenetetrahydrofolate dehydrogenase 1/2/1L; MTHFD1/2/1L, which supplies its 1C unit for purine synthesis [28].

### 2.3. Oxidative Phosphorylation

One of the key functions of mitochondria is oxidative phosphorylation (OXPHOS), by which ATP, the principal source of cell energy, is generated through the ETC [29]. Reducing equivalents nicotinamide adenine dinucleotide (NADH) and flavin adenine dinucleotide (FADH_2_), produced in the TCA cycle, are necessary to transfer electrons to the ETC, which takes place in the inner mitochondrial membrane, where enzymatic multiprotein complexes participating in the process are located (complexes I through V) [30]. NADH and FADH_2_ transfer electrons to complex I and complex II, respectively, from which they are transferred to ubiquinone (Q) and further to complex III [30]. From complex III, electrons are passed to cytochrome c, which reduces oxygen into water in the presence of protons [30]. During this sequential transfer of electrons through various complexes, protons are transferred to the intermembrane space of the mitochondria, inducing the generation of a large mitochondrial membrane potential (Δψ_m_) [8]. Finally, the complex V (ATP synthase) is responsible for pumping the protons back into the mitochondrial matrix via the F_0_ subunit, thus releasing the energy of Δψ_m_, which is used by the F_1_ subunit of ATP synthase to catalyze the phosphorylation of adenosine diphosphate (ADP) into ATP [30].

It is important to note that the mitochondrial ETC also underlies the production of ROS [31]. This is mainly due to the fact that some electrons escape from the ETC and react with oxygen directly, causing its mono-electronic reduction, resulting in the formation of the superoxide anion radical (O_2_^−^) [32]. In the presence of such electron leakage, O_2_^−^ is usually produced by complexes I and III [31]. Under physiological conditions, O_2_^−^ is readily transmuted to hydrogen peroxide (H_2_O_2_) in a reaction catalyzed by mitochondrial manganese superoxide dismutase (MnSOD), which is further transformed into water by mitochondrial glutathione peroxidase (GPx) in the presence of reduced glutathione (GSH). The non-detoxified H_2_O_2_, in normal and acute conditions, can diffuse into the cytoplasm, where it acts as a second messenger and can modulate gene transcription, induce hypoxia-inducible factor-1 (HIF-1) stabilization, and therefore have a protective effect on hepatocytes regarding oxidative damage and apoptosis [33,34,35,36]. On the other hand, in chronic liver injury, this can lead to steatosis, fibrosis, and hepatocellular carcinoma progression [37,38,39]. Moreover, H_2_O_2_ can serve as a substrate in the Fenton reaction in the presence of Fe^2+^ and can be transformed into a hydroxyl radical (HO·), which can cause lipid peroxidation as well as protein and DNA oxidation [19]. In conditions of extensive stress for the liver, such as upon chronic ethanol consumption, fasting, or malnutrition, mitochondrial GSH levels can be depleted, thus compromising the H_2_O_2_ detoxification process and favoring its accumulation [8]. Furthermore, in the case of the impaired flow of electrons in the ETC, as well as in the presence of excessive ETC substrate supply and electron overload without the dissipation of Δψ_m_ by the ATP synthase, the increased production of ROS can occur [19,40]. Constantly increased levels of ROS can negatively affect mitochondrial proteins involved in OXPHOS and mtDNA, hence contributing to mitochondrial dysfunction, electron leakage from the ETC, and further ROS production and oxidative damage [41,42]. Of note, mtDNA is extremely sensitive to oxidative injury due to a lack of all necessary mtDNA repair enzymes, the absence of histones, and the proximity to the inner mitochondrial membrane, which is the main source of ROS [8].

### 2.4. Mitochondrial Permeability Transition Pore/Mitochondrial Outer Membrane Polarization and Cell Death

Mitochondrial membrane integrity is of critical importance for maintaining proper mitochondrial function. However, in certain conditions, mitochondrial membrane permeability can be disrupted. This can be due to the opening of the mitochondrial permeability transition pore (MPTP) or involving the peripheral benzodiazepine receptor (PBR), the voltage-dependent anion channel, adenine nucleotide translocase, and cyclophilin D [43]. Such disruptions of MPTP lead to alterations in mitochondrial function and structure, and to cell death [43]. MPTP opening can significantly affect ATP synthesis, ATP levels, and intracellular calcium levels resulting in necrosis [44]. In addition, MPTP opening can cause mitochondria to swell and membrane rupture, allowing the release of pro-apoptotic factors and apoptosis initiation [12]. Furthermore, mitochondria can also mediate the intrinsic pathway of apoptosis via MOMP and pro-apoptotic members of the Bcl-2 family of proteins, including Bax and Bak [12]. In the presence of stress stimuli, such as DNA damage and a lack of mitogenic signals, MOMP occurs followed by the release of various factors from the mitochondrial intermembrane space, which triggers apoptosis [12]. Once released in the cytoplasm, cytochrome c binds the adaptor molecule apoptotic peptidase activating factor 1 (APAF1), forming the apoptosome, which subsequently activates the initiator caspase 9 via proteolytic cleavage [12,45]. Finally, caspase 9 cleaves and activates the executioner caspases 3 and 7 responsible for apoptosis [12]. Under pro-apoptotic stimuli, Bax and Bak are activated and homodimerize to form oligomers that can form lipidic pores in the outer mitochondrial membrane followed by the release of soluble intermembrane space proteins, such as cytochrome c, Smac, and Omi [12,46,47]. These proteins can block the X-linked inhibitor of apoptosis protein (XIAP), thereby facilitating apoptosis. When Bax/Bak-induced pores expand over time, they create macropores that allow the extrusion of the inner mitochondrial membrane through the outer mitochondrial membrane, and the release of mtDNA in the cytoplasm upon its rupture [12]. Released mtDNA is responsible for triggering various innate immune and pro-inflammatory responses, including the type I interferon response [48]. The extrinsic pathway of apoptosis converges into the intrinsic one, specifically where caspase 8 cleaves Bid, a pro-apoptotic BH3-only Bcl-2 family member, generating tBid that subsequently induces MOMP [12].

## 3. Mechanisms of Liver Injury

Liver injury is manifested as morphological and functional damage that can be caused by various biological and chemical agents. Many of those harmful agents are easily accessible to humans, being the products of food, pharmaceutical, and chemical industries, but also because they can be found in nature as products of animal, plant, fungal or bacterial metabolism, or be present in the environment as industrial waste products and pollutants [49]. There are many factors influencing the type and extent of toxin-mediated liver injury. These include the physicochemical characteristics of the toxic agent, the mechanisms of toxicity, the nature of exposure, the efficacy of hepatocellular detoxification systems, genetic polymorphisms affecting metabolic and transport pathways, and various (patho)physiological conditions [49,50]. All these factors can impair liver function or increase susceptibility to damage [49]. In severe cases and conditions where the exposure to a hepatotoxin is not interrupted, extensive damage leading to liver failure can occur. Liver damage can result either from direct insult to the hepatocytes or from damage to other liver cell types, including stellate cells, Kupffer cells, sinusoidal epithelial cells, and bile canalicular cells, which can indirectly affect hepatocytes or cause their injury [51]. There are different types of liver damage, in particular cholestatic and hepatocellular insults, as well as their combinations, all of which involve different mechanisms [49].

### 3.1. Cholestatic Injury

Cholestatic injury relates to the impairment of bile flow or secretion. Cholestasis is commonly caused by drugs or their metabolites following the inhibition of hepatobiliary transporter systems necessary for bile formation and secretion [52]. This leads to increased concentrations of noxious bile acids [53]. The main mechanism of bile acid-induced hepatotoxicity is hepatocellular necrosis [54,55]. Moreover, at the critical micellar concentration, bile acids act as detergents, causing plasma membrane damage, thus inducing hepatocyte injury [56]. Cholestasis is clinically diagnosed based on increased levels of bilirubin, alkaline phosphatase (ALP), and gamma-glutamyl transpeptidase (GGT) [49].

### 3.2. Hepatocellular Injury

Hepatocellular injury can occur through various pathways, such as direct, immune, metabolism-related, and mitochondria-mediated toxicity [57].

#### 3.2.1. Direct Hepatotoxicity

Direct hepatotoxicity is usually attributable to apoptosis or necrosis as the main mechanisms [53]. Apoptosis can occur via the extrinsic pathway triggered by the activation of death receptors, such as Fas, tumor necrosis factor-related apoptosis-inducing ligand (TRAIL) and tumor necrosis factor receptor 1 (TNFR1), and the intrinsic mitochondrial pathway, activated by intracellular perturbations, such as DNA damage, lysosomal permeabilization, endoplasmic reticulum stress, oxidative damage and increased levels of calcium [44]. Even though apoptosis is a tightly controlled and programmed cell death form causing no harm to healthy cells, it can lead to liver injury through excessive inflammation [58]. On the other hand, during necrosis, usually triggered by ATP depletion or other massive noxious stimuli, there is an uncontrolled cell swelling and rupture with the subsequent release of endogenous danger-associated molecular patterns (DAMPs) that induce inflammation and the innate immune system, which further contributes to tissue damage [44]. Hepatocellular injury is clinically often detected based on serum aminotransferases, in particular alanine aminotransferase (ALT or ALAT) and aspartate aminotransferase (AST or ASAT) [49].

#### 3.2.2. Immunological Hepatotoxicity

The immunologic hepatotoxicity is mediated by the covalent complexes, haptens, formed between the toxin or its intermediates and cellular proteins. Haptens are highly immunogenic and act as antigens, therefore eliciting excessive or inappropriate immune responses [59,60]. They are able to induce the activation of antigen-presenting cells, which activate T cells that exert cytotoxic activity towards hepatocytes [60]. Moreover, DAMPs released during hepatocyte injury can further amplify the hapten-induced immunogenic signal, therefore initiating the actual immune intolerance characterized by prominent T-cell cytotoxicity and cell death [59,60]. In addition to haptens, some drugs can directly interact with the immune system by binding to the highly variable antigen-specific regions of T cells and inducing their activation, which results in prolonged and potent immune responses leading to extensive liver damage [61,62].

#### 3.2.3. Metabolism-Related Hepatotoxicity

Cytochrome P450 (CYP) isoenzymes play an important role in metabolism-related hepatotoxicity. In fact, they can activate or metabolize some chemicals and drugs to reactive intermediates that can have multiple direct and indirect toxic effects [50,63]. The most important CYP isoenzymes responsible for xenobiotic metabolism, and therefore in this type of hepatotoxicity, are CYP1A1, CYP1A2, CYP1B1, CYP2A6, CYP2C9, CYP2E1, and CYP3A4. Such reactive intermediates can interact and covalently bind to cellular macromolecules, including nucleic acids, proteins, and lipids, affecting their structure and function, and thus altering various cellular processes [64]. They can also affect cellular antioxidant defense mechanisms by depleting GSH and even mediate lipid peroxidation. As a result, many cellular organelles are directly affected and functionally compromised. In particular, by interacting with membrane lipids, the reactive metabolites can severely affect the permeability of the plasma membrane, endoplasmic reticulum, and mitochondrial membranes, leading to alterations in calcium homeostasis and subsequent cell damage [64]. Necrosis due to ATP depletion has also been described as a mechanism in this type of hepatotoxicity [7]. These metabolic derivatives can induce indirect toxic effects via the regulation of signal transduction pathways and gene expression profiles, leading to cell death, either by apoptosis or necrosis [65]. However, this type of hepatotoxicity can occur if the toxin in question is acutely present at extremely high concentrations or lower concentrations over long periods of time [50]. However, it is worth mentioning that this type of liver toxicity is very complex, considering that there are numerous developmental, genetic, and environmental factors that can significantly influence CYP-mediated drug metabolism and response, and consequently their potential toxic effects [50]. This is particularly true for drugs with a narrow therapeutic window, where inter-individual differences and genetic polymorphisms are crucial factors to be considered to avoid toxicity when determining dose regimen [50].

#### 3.2.4. Mitochondria-Mediated Hepatotoxicity

The mitochondrial type of hepatocellular injury commonly exhibits alterations of lipid metabolism, OXPHOS, and the depletion of ATP, which cause lactic acidosis and microvesicular steatosis, but also the altered activity of the enzymatic complexes in the ETC, MnSOD, and GPx, leading to excessive ROS generation and oxidative damage [66,67].

## 4. Drug-Induced Mitochondrial Dysfunction and Liver Injury

**Figure 3:**
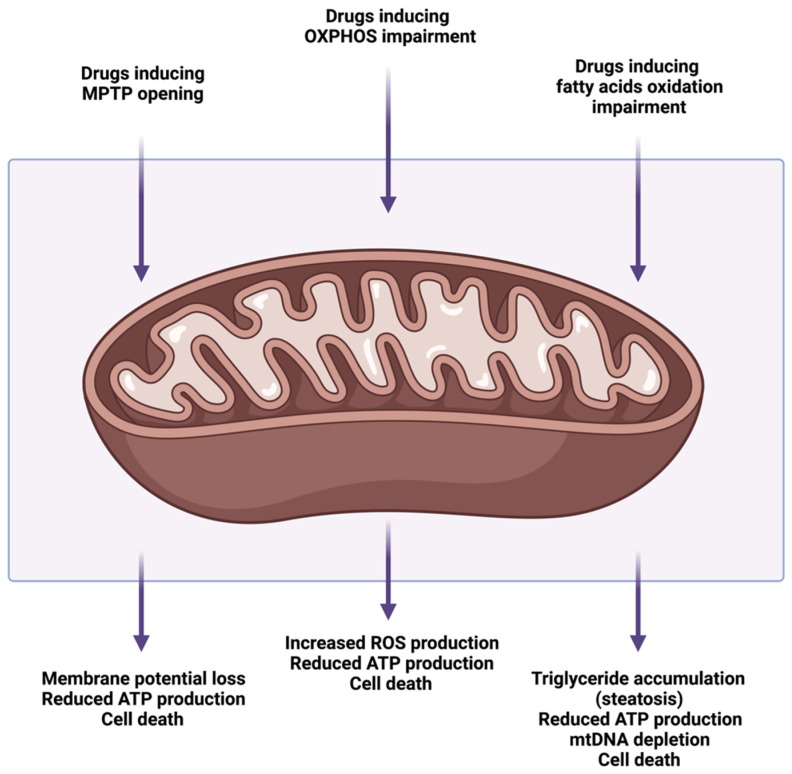
Schematic representation of the main mechanisms and consequences of drug-induced mitochondrial dysfunction. Created with Biorender.com (accessed on 21 February 2022; Toronto, ON, Canada). (MPTP—mitochondrial permeability transition pore; OXPHOS—oxidative phosphorylation; ATP—adenosine triphosphate; ROS—reactive oxygen species; mtDNA—mitochondrial DNA).

Drug-mediated mitochondrial toxicity represents one of the main mechanisms of DILI. Not surprisingly, many of the drugs reported to interfere with mitochondrial function cause symptoms similar to those of patients suffering from (genetic) mitochondrial diseases [68]. There are various mechanisms by which hepatotoxic drugs can cause mitochondrial dysfunction. These include the direct drug-induced inhibition of mitochondrial function, drug interference with mtDNA, with transcription and protein synthesis, mitochondrial dysfunction mediated by drug-derived reactive metabolites, and mitochondrial injury due to the increased susceptibility of specific groups of patients (Figure 3). Specifically, drugs capable of inducing mitochondrial toxicity can cause membrane polarization, the impairment of OXPHOS, or the impairment of fatty acid oxidation, by affecting different targets [8]. It is important to note that the events leading to mitochondrial toxicity are extremely complex, whereby different targets can lead to the same outcomes, such as steatosis. For instance, the inhibition of fatty acid enzymes, the disruption of the ETC, and mtDNA depletion can all lead to steatosis [6,8]. Besides, the same drugs can affect mitochondrial function at different points, such as valproic acid, which causes oxidative stress, leading to MPTP opening, but also inhibits fatty acid enzymes, therefore causing steatosis [69,70,71,72].

### 4.1. Mitochondrial Permeability Transition Pore Opening

Various drugs are capable of inducing MPTP opening in liver mitochondria, thus causing cytolytic hepatitis characterized by extensive apoptosis and necrosis, which leads to hepatic failure [4]. Depending on the extent of the damage, increased ALT, AST, and LDH plasma levels can be measured, even though these markers cannot be considered specific to mitochondrial hepatotoxicity [8]. Despite not being fully elucidated, there are different mechanisms proposed by which these drugs induce MPTP opening (Table 1). Some drugs can directly interact with PBR, an MPTP component, thereby inducing mitochondrial membrane permeabilization and subsequent cell death [73]. Other drugs can indirectly cause MPTP opening and the release of pro-apoptotic proteins via the activation of c-Jun N terminal protein kinase (JNK), followed by the cleavage of Bid and the release of cytochrome c from mitochondria. Moreover, some drugs have been shown to indirectly affect the excessive production of ROS, which oxidize thiol groups of membrane proteins involved in regulating MPTP [74], while others interfere with iron metabolism, which has also been shown to induce MPTP [75]. In particular, lysosome instability allows ferrous iron translocation into mitochondria, most likely through the mitochondrial electrogenic Ca^2+^/Fe^2+^ uniporter, which causes MPTP opening and mitochondrial dysfunction [76]. As a consequence of the MPTP opening, loss of membrane potential and decreased ATP production trigger cell death by necrosis [7].

### 4.2. Alterations of Oxidative Phosphorylation and Electron Transport Chain

Certain drugs are also able to interfere with OXPHOS and ETC leading to reduced ATP synthesis and its depletion, or a severely affected ETC with subsequent altered oxidation processes [68]. Depending on the drug and/or its concentration, several mechanisms of alteration in OXPHOS can be distinguished (Table 2). One of the mechanisms is OXPHOS uncoupling without the inhibition of ETC, which causes a significant reduction in ATP synthesis [87]. Drugs frequently observed as being capable of such an action are cationic amphiphilic molecules that can be protonated in the mitochondrial intermembrane space and subsequently transported into the mitochondrial matrix through the Δψ_m_ [88]. This reversal of the proton flux from the intermembrane space into the matrix, while bypassing the ATP synthase, dissipates the proton gradient generated during electron transport, which ultimately disrupts the OXPHOS and drastically reduces ATP synthesis [88]. Another mechanism includes OXPHOS uncoupling with the inhibition of ETC, which also further impairs oxidation processes, including fatty acid β-oxidation [68,89]. The inhibition of ETC by OXPHOS uncouplers is thought to be concentration-dependent, and even though the exact mechanisms might not be fully elucidated, some data suggest that the direct inhibition of ETC complexes is responsible for ETC impairment [90,91]. Alterations in OXPHOS can also be due to a blockage of ETC by the direct inhibition of ETC complexes and without prior OXPHOS uncoupling [92].

### 4.3. Alterations of Mitochondrial Fatty Acids β-Oxidation

Various drugs can directly or indirectly target mitochondrial fatty acid oxidation, leading to hepatocyte damage [6,68,89]. The main consequence of impaired fatty acid oxidation is the intracellular accumulation of triglycerides within hepatocytes and, in extreme conditions, free fatty acids build-up [89]. The most common types of intracellular lipid accumulation, occurring as a result of DILI, are microvesicular (multiple intracellular tiny lipid droplets) and macrovesicular (single intracellular large lipid vacuole) steatosis, which can be present either individually or co-existing [89,104,105]. While the former is a much more severe hepatic lesion that can be possibly associated with liver failure, encephalopathy, hypoglycemia, and even coma and death, the latter is more common and considered to be a benign lesion in the short term, though after a prolonged time it can lead to complications such as steatohepatitis and, rarely, fibrosis [89,104,106,107,108,109,110]. Other consequences of impaired β-oxidation are reduced energy production (ATP shortage) and cell death, and related to that, hypoglycemia-inducing reduced gluconeogenesis due to reduced levels of acetyl-CoA and the impaired activity of pyruvate carboxylase [89]. Furthermore, the accumulation of fatty acid derivatives in plasma and urine can occur because of impaired β-oxidation [89]. Several mechanisms have been described to result in drug-induced alterations of mitochondrial β-oxidation (Table 3). Some drugs affect fatty acid oxidation by inhibiting enzymes, such as CPT1 and acyl-CoA synthases, thereby interfering with and affecting multiple points of the oxidation process [70,111]. Drugs can impair mitochondrial fatty acid oxidation by decreasing the levels of important cofactors, including CoA and L-carnitine esters [112,113,114]. The significant inhibition of the mitochondrial ETC can also lead to impaired β-oxidation. Some drugs can act via a dual mechanism, namely the inhibition of fatty acid oxidation enzymes at lower concentrations and the impairment of ETC at higher concentrations [91,98,115,116]. Drugs can equally cause a depletion of mtDNA, therefore affecting ETC and subsequently β-oxidation. Although the exact mechanisms by which drugs reduce mtDNA levels are not completely understood, evidence suggests that interactions with mitochondrial topoisomerases negatively affect mtDNA synthesis and levels [100,117,118,119,120,121,122,123]. Drug-induced ROS production and excessive oxidative stress have been reported to induce mtDNA strand breaks and damage, ultimately resulting in a reduction of mtDNA levels [124,125].

## 5. Factors Influencing Drug-Induced Hepatic Mitochondrial Dysfunction

**Figure 4:**
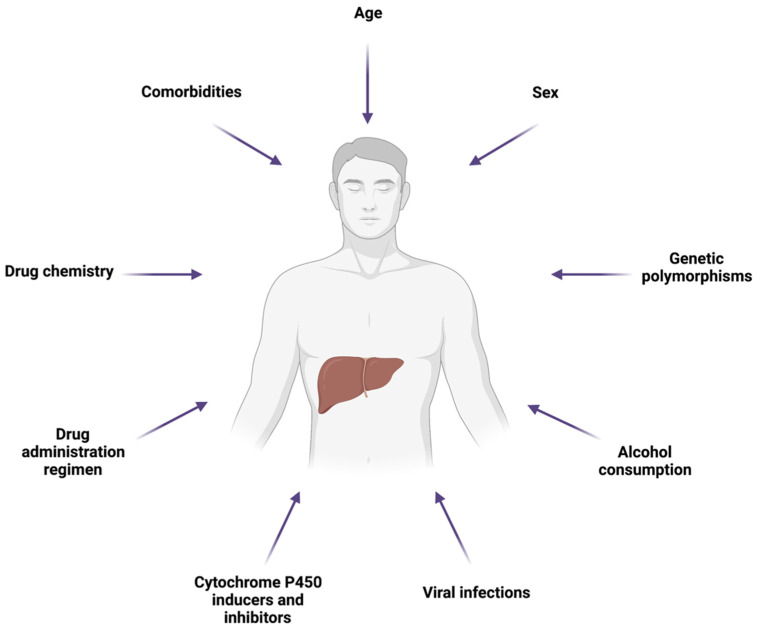
Schematic representation of the main factors affecting susceptibility to hepatic injury due to drug-induced mitochondrial toxicity. Created with Biorender.com (accessed on 21 February 2022; Toronto, ON, Canada).

A plethora of specific conditions and factors can predispose to or aggravate hepatic injury due to mitochondrial toxicity, including drug chemistry and administration regimen, genetic polymorphisms, variation in mtDNA, non-genetic host factors, comorbidities, and external factors, including environment and lifestyle (Figure 4) [8].

The chemical structure of the drugs is very important and can be accountable for mitochondrial toxicity. In this respect, amphiphilic molecules that possess protonable moieties can be transported to and accumulate within the mitochondrial matrix due to Δψ_m_. Consequently, vital processes, such as OXPHOS, are impaired [88,91,98,115]. On the other hand, drugs that have a fatty acid structure, such as the branched-chain fatty acid valproic acid, can be activated by CoA, and therefore account for the sequestration of this important cofactor and subsequent impaired β-oxidation [112]. Furthermore, nucleotide reverse transcriptase inhibitors (NRTIs) can be incorporated within mtDNA by the mtDNA polymerase γ and inhibit mtDNA replication, causing its depletion and affecting vital mitochondrial processes [119].

Drug dose and duration regimen are also key factors in influencing hepatic mitochondrial dysfunction and DILI. A number of studies have suggested that tetracycline-associated microvesicular steatosis is dose-dependent and that prolonged treatment with amiodarone can induce liver injury even after therapy discontinuation, which is linked to its accumulation in different tissues, including liver, lung and adipose tissue [68,140,141].

Genetic predisposition is a major factor affecting the susceptibility of developing mitochondrial hepatotoxicity. DNA mutations and polymorphisms can significantly increase the risk of DILI, by affecting normal mitochondrial function, the activity of drug-metabolizing enzymes, and enzymes involved in oxidative stress defense. Individuals suffering from cytochrome c oxidase deficiency or a deficiency of medium-chain acyl-CoA dehydrogenase, which is involved in the mitochondrial oxidation of medium-chain fatty acids, are more prone to develop DILI associated with drugs that impair mitochondrial function, such as valproic acid [142,143,144]. Several polymorphisms in CYP genes, including CYP2C9, CYP2C19, CYP2D6, CYP2E1, and CYP2B6, have been associated with DILI, including steatosis, steatohepatitis, and cirrhosis [145,146,147,148]. The deletion of glutathione S-transferase theta 1 (GSTT1) and glutathione S-transferase Mu 1 (GSTM1), involved in the detoxification and prevention of oxidative stress, have also been reported to increase the risk of hepatotoxicity [145,149,150,151,152]. MnSOD is another enzyme essential for cellular defense mechanisms against oxidative stress, and its genetic polymorphisms have also been linked with increased susceptibility to mitochondrial toxicity by various drugs [67,153,154,155,156,157].

There is a large inter-individual variation in mtDNA copy number, and while the origin of this variation is still unknown, low mtDNA levels could present a risk factor for the mitochondrial toxicity of drugs known to interfere with mtDNA [6]. In addition, variations and mutations in mtDNA represent an important inter-individual difference associated with adverse drug reactions and idiosyncratic DILI [158,159]. Emerging studies have shown that mitochondrial genetics and specific mtDNA haplogroups are involved in increased susceptibility to drug toxicity, especially for toxicity induced by antibiotics, antiretrovirals and chemotherapeutic agents [159].

Moreover, non-genetic host factors that majorly influence DILI are age and sex. Age is considered a risk factor depending on the drug, with certain age populations being more vulnerable to specific drugs [160]. In this regard, young children are at risk of hepatotoxicity due to valproic acid and aspirin [160]. However, the elder population is more susceptible to DILI caused by numerous drugs, such as erythromycin, isoniazid, and amoxicillin [160,161,162]. While the reasons for the age-dependent DILI are not known, available research suggests that altered drug pharmacokinetics due to reduced renal function, reduced liver blood flow, and reduced CYP-mediated metabolism, as well as the increased production of reactive intermediates and co-medication in elder people, could play a role [163,164,165,166]. Gender has also been shown to affect the risk of DILI, with women and men having different degrees of susceptibility, due to different metabolism efficiency [166]. Sex hormones, pregnancy, and growth hormone levels can influence drug metabolism. Thus, men have higher glucuronidation rates and therefore a more efficient clearance rate of acetaminophen, while women possess higher expression levels of CYP3A4 [167,168].

Other recognized predisposing factors for mitochondrial toxicity and DILI are underlying comorbidities, including obesity, non-alcoholic fatty liver disease (NAFLD), and type 2 diabetes. In fact, increased susceptibility to liver injury and the aggravation of hepatic lesions in obese and NAFLD patients could be attributable to underlying mitochondrial dysfunction (latent ETC dysfunction), reduced antioxidant defenses (low GST expression and GSH levels), the enhanced expression and activity of CYP isoenzymes (CYP2E1), and pro-inflammatory and pro-fibrotic cytokine production (tumor necrosis factor α; TNF-α) [169,170,171,172,173].

Some of the key environmental factors that can increase vulnerability to drug-induced hepatic mitochondrial toxicity are alcohol consumption, CYP inducers and inhibitors, and viral infections.

Excessive ethanol consumption can have deleterious effects on hepatic mitochondrial function, and by causing mitochondrial dysfunction, it renders the liver more prone to DILI [174,175]. Moreover, ethanol is metabolized by hepatic CYP2E1 and its overconsumption increases CYP2E1 levels in mitochondria, hence affecting the metabolism of numerous drugs and enhancing the formation of reactive metabolites that can directly cause mitochondrial dysfunction [7,176]. Furthermore, drugs can induce other CYP isoenzymes, such as CYP1A1 and CYP2B1. By doing so, they significantly affect hepatic drug metabolism and increase the generation of reactive hepatotoxic intermediates [177,178,179]. In addition, ethanol intoxication can induce SREBP-1c activation, thus stimulating hepatic lipogenesis [180].

Many drugs, food components, herbal products, and pollutants are known to modulate CYP isoenzymes and thereby affect drug metabolism and DILI. The most affected are CYP3A4, CYP3A5, CYP1A2, CYP2B6, CYP2C8, CYP2C9, CYP2C19, CYP2D6, CYP2A6, and CYP2E1 [181]. The compounds shown to interfere with their function comprise many drug classes, such as antiepileptics, antiretrovirals, antibiotics, antimalarials, barbiturates, proton pump inhibitors, glucocorticoids, and many others [181]. Moreover, nutritional and herbal compounds capable of such effects include, but are not limited to, resveratrol, quercetin, theophylline, caffeine, hyperforin, genistein, baicalin, sulforaphane, and indole-3-carbinol [181]. Moreover, there are numerous other toxic agents that can modulate drug-metabolizing enzymes, including dioxins, polycyclic aromatic hydrocarbons, heterocyclic aromatic amines, organochlorine pesticides, polychlorinated biphenyls, benzene derivatives, and others [181].

The hepatitis C virus is a recognized contributing factor for drug-induced mitochondrial dysfunction [6]. Viral infections can increase oxidative stress and the release of pro-inflammatory factors, therefore impairing both mitochondrial function and lipid homeostasis, and considerably increasing the risk of drug-induced mitochondrial toxicity [6]. This has been reported for various drugs, and in particular for aspirin, shown to increase the occurrence of Reye’s syndrome in the presence of an ongoing viral infection, as well as for NRTIs that can drastically increase susceptibility to hepatotoxicity in individuals infected with the hepatitis C virus [182,183,184,185]. It is thought that viral proteins, together with pro-inflammatory cytokines and the oxidative stress generated during infection, affect mitochondrial function and potentiate mitochondrial toxicity and liver injury by NRTIs [6,183].

## 6. Experimental Models and Methods to Study Hepatic Mitochondrial Toxicity

### 6.1. Experimental Systems and Models to Study Mitochondrial Dysfunction and Related Hepatotoxicity

There are numerous in vitro tools that are largely implemented for the assessment of the hepatotoxic potential of various compounds and that offer a valid platform to investigate mechanisms of mitochondrial toxicity. Human-based models are preferred over animal-derived systems because of their better predictive and translational value [186,187]. However, both are used, especially since there is a low availability of human material.

Many in vitro assays make use of isolated liver mitochondria, which represent an essential tool to study not only mitochondrial structure and functions but also compounds capable of inducing mitochondrial dysfunction [188]. The isolation of liver mitochondria is usually carried out by differential centrifugation and allows one to obtain functional, intact, and relatively pure organelles that can be used to study various mitochondrial parameters in situ, both under physiological conditions and in the presence of underlying pathological conditions or toxic compounds [189]. Mitochondria can be isolated from different tissues such as liver, muscle, and kidney tissue as well as cultured cells [189,190]. However, isolated mitochondria present different limitations as well. Besides the obvious lack of cellular context, there are certain issues related to the isolation method and source material that can affect mitochondrial function. These include the necessity of a large amount of tissue sample for successful isolation, high mitochondrial susceptibility to damage, alterations in ETC complex subunits, and increased ROS production during centrifugation [191,192].

Next to the isolated mitochondria, hepatocyte culture systems are yet another highly valuable tool to study mitochondrial toxicity and dysfunction. Despite being used successfully for mitochondrial dysfunction studies and representing the gold standard and most relevant model from the clinical translational point of view, primary human hepatocytes do have some limitations [193,194,195]. These include inter-individual variability, scarcity, susceptibility to dedifferentiation, and low quality, considering that the source of hepatocytes is usually liver biopsies obtained from individuals suffering from liver disease or material unsuitable for liver transplantation [193,196,197,198,199]. Therefore, other cell culture models have been used widely as alternatives, including primary rat and mouse hepatocytes, which under certain circumstances are acceptable for mitochondrial toxicity evaluation [93,200,201]. By far the most popular culture system is represented by human hepatoma cell lines, such as HepG2, Hep3B, Fa2N4, HepaRG, and Huh7 cells [193]. These cell lines, despite not being completely physiologically representative, show genetic instability and reduced expression levels of biotransformation enzymes, offer high reproducibility, are readily available, simple to culture, and suitable for mitochondrial toxicity testing [193,202,203]. An additional crucial limitation of such cell lines, compared to primary cells, is their altered bioenergetic phenotype. These cells are metabolically adapted to grow in acidic and hypoxic conditions, relying mostly on glycolysis to obtain the energy [204]. Despite possessing fully functional mitochondria, they do not obtain the energy via OXPHOS, which significantly reduces the predictive value when studying mitochondrial toxicants [205,206]. This has led to the development of the “glucose-galactose” assay based on HepG2 cells, in which the cells are grown in the presence of galactose [205]. This induces slow glycolytic conditions and higher cell dependency on mitochondrial OXPHOS for energy production, which makes them more vulnerable to drugs targeting mitochondria and more suitable for the evaluation of drug-induced mitochondrial dysfunction [205,206,207].

Furthermore, there are numerous animal models used to study DILI and related hepatotoxicity mechanisms, with rodents being undoubtedly preferred due to higher accessibility and easier experimental implementation [208,209]. However, different animal species have been used to study DILI-related mitochondrial dysfunction, including dogs, rabbits, and primates [6]. In addition to wild-type animals, hepatic mitochondrial toxicity can be studied in certain genetic models, such as the heterozygous MnSOD^+/−^ knockout mouse [156,210]. This model has underlying liver mitochondrial problems, such as decreased Δψ_m_ and decreased ETC activity, and can reveal drug-induced mitochondrial dysfunction not detectable in wild-type models [211,212].

### 6.2. Experimental Methods and Assays to Study Mitochondrial Dysfunction and Related Hepatotoxicity

Drug-induced mitochondrial dysfunction can be assessed in isolated mitochondria, cells (cell lines, cells derived from in vivo models of mitochondrial toxicity), or liver tissue, by using numerous methods that can evaluate the capacity of a drug to trigger MPTP opening, interfere with fatty acid oxidation, uncouple or inhibit OXPHOS, and cause mtDNA damage and depletion and DILI-induced liver lesions (Table 4).

MPTP opening, known to be involved in drug-induced mitochondrial toxicity, can be determined experimentally using the mitochondrial swelling assay [215,292]. This method is based on measuring spectrophotometrically mitochondrial swelling and conformational changes, direct consequences of MPTP opening, which are reflected in a decrease in absorbance at optical density in the presence of Ca^2+^ [214]. Moreover, MPTP can also be assessed by measuring the ability of mitochondria to take up and retain extramitochondrial Ca^2+^. For this purpose, Ca^2+^-sensing fluorescence dyes are used to reflect Ca^2+^ uptake. The fluorescence hereby decreases as added Ca^2+^ is taken up by mitochondria until MPTP occurs [214]. In addition, microscopy techniques (fluorescence, confocal or electron microscopy) can be used to visualize and measure mitochondrial swelling in cells and tissue samples [216].

Fatty acid oxidation can be assessed both in isolated mitochondria and cells by using radio-labeled fatty acids, such as ^14^C-labeled palmitate [219]. The ^14^C label can be present at different positions in the palmitate, depending on whether complete or incomplete oxidation is measured [219]. During fatty acid oxidation, ^14^C-palmitate can be oxidized to different acid-soluble metabolites containing the ^14^C label, such as palmitoyl-carnitine, acetyl-carnitine, acetyl-CoA, ketone bodies, fatty acyl-CoA shorter than 6 carbons, gluconeogenic and TCA cycle intermediates, as well as ^14^C-carbon dioxide (^14^CO_2_), which is the product of radiolabeled acetyl-CoA entering the TCA cycle [219]. Finally, the rate of conversion of ^14^C-palmitate can be calculated for any of the acid-soluble metabolites or ^14^CO_2_ produced [219]. In in vivo models, ^14^C-labeled fatty acids can be administered and exhaled ^14^CO_2_ measured to determine whole-body fatty acid oxidation [136]. The use of different lengths of ^14^C-labeled fatty acids allows one to assess whether the whole fatty acid oxidation process is affected or only processes regarding specific chain length [136]. Therefore, this type of assay allows one to precisely monitor the complete oxidation process and determine the efficiency and possible modulation of fatty acid oxidation [219].

An indirect measure of mitochondrial dysfunction, linked to altered fatty acid oxidation, is the assessment of steatosis. The presence of lipid droplets in the cells following drug-induced mitochondrial injury can be evaluated by means of one of the several available staining procedures, including Oil Red O, Sudan Black B, Nile Red, and 4,4-difluoro-1,3,5,7,8-pentamethyl-4-bora-3a,4a-diaza-s-indacene (BODIPY^TM^ 493/503) [293,294,295,296]. These are often detected with microscopy techniques or flow cytometry. Absolute lipid quantification assays are also used to determine intracellular lipid levels, in which various multi-step protocols are used to extract and quantify the total lipid content, either with high-performance liquid chromatography (HPLC) and liquid chromatography-mass spectrometry (LC-MS) methods or fluorimetric-based and colorimetric-based assays [297,298,299,300].

Energy metabolism and the efficiency of OXPHOS are usually assessed by measuring the oxygen consumption rate (OCR) and can be done using isolated mitochondria, suspensions of cells, or small amounts of tissue in a specific cell culture media [301]. OCR is calculated by determining the rate of decrease in oxygen concentration in the cell culture medium by a polarography electrode [231]. Recent variations of this method allow OCR measurement to be performed in adherent cells in situ and with reduced sample volume by using optical techniques and the oxygen-mediated quenching of phosphorescence or fluorescence [302,303]. Moreover, novel methods, such as the Seahorse XF Extracellular Flux Analyzer (Agilent Technologies) and the Oroboros Oxygraph-2k System (Oroboros Instruments), can be efficiently used in real-time for the assessment of OCR [233,234].

Mitochondrial OXPHOS performance can be evaluated by assessing the enzymatic activity of respiratory chain complexes, which is commonly done by means of spectrophotometric assays [249]. Complex I activity is measured by monitoring the oxidation of NADH at 340 nm, detectable as a decrease in absorbance [249]. Complex II activity is measured by monitoring the reduction in ubiquinone with absorbance at 280 nm or the oxidation of electron acceptor 2,6-dichlorophenolindophenol with absorbance at 600 nm [249]. The combined activity of complexes II and III is measured by determining the reduction in cytochrome c with an increase in absorbance at 550 nm [249]. Furthermore, the activity of complex IV can be measured by monitoring the oxidation of cytochrome c and the subsequent decrease in absorbance at 550 nm [249]. Finally, complex V activity is based on the measurement of the reverse ATP hydrolysis reaction, coupled to NADH oxidation, and the conversion of phosphoenolpyruvate to pyruvate, by pyruvate kinase, and pyruvate to lactate, by LDH, by monitoring changes in absorbance at 340 nm [249,304]. In addition, the protein expression of the respiratory chain complexes, detected by immunoblot analysis, can be used complementarily to their enzymatic activity when determining mitochondrial OXPHOS function.

Measurements of mitochondrial cofactors NADH and nicotinamide adenine dinucleotide phosphate (NADPH), crucial for enzymatic redox reactions, is also a valuable indicator of mitochondrial function. The changes in their levels can indicate increased ETC activity (increased NADH oxidation), decreased TCA cycle activity (decreased NAD^+^ reduction), or increased NAD^+^ consumption [305]. The autofluorescence of NADH and NADPH is usually exploited to determine their levels and it can be detected using confocal microscopy (excitation wavelength 340–360 nm; emission wavelength 450 nm) or fluorescence lifetime imaging microscopy [237]. HPLC and spectrometry techniques are also used to monitor these cofactors [306]. Moreover, genetically encoded fluorescent proteins, such as Rex protein, circularly permuted yellow fluorescent protein, and Peredox, can bind to NADH and NAD^+^, and therefore can be measured via fluorescence-based methods to determine their levels and ratios [240,241,307]. The measurement of mitochondrial NADH and NADPH is commonly performed together with Δψ_m_ measurements for more reliable monitoring of mitochondrial activity [251].

Another important in vitro tool used to detect mitochondrial dysfunction is the determination of Δψ_m_. Among the most frequently used assays for Δψ_m_ assessment is the use of cationic cell membrane-permeable fluorescent dyes, including tetramethyl rhodamine methyl and ethyl esters (TMRM, TMRE), rhodamine-123 (Rh123), 3,3′-dihexyloxacarbocyanine iodide (DiOC6(3)), 1,1′,3,3′-tetraethyl-5,5′,6,6′-tetrachloroimidacarbocyanine iodide (JC-1), MitoTracker^TM^ Red CMXRos, and safranine, all of which are selective for the mitochondria of live cells and reflect changes in Δψ_m_, which can be detected using flow cytometry, fluorescence imaging, or spectrofluorimetry [244,308]. In addition, Δψ_m_ can be measured by using tetraphenylphosphonium cation (TPP(+))-selective electrodes, which allows one to assess the concentration of this mitochondria-permeable probe in the cell culture medium, thus reflecting changes of TPP(+) mitochondrial accumulation and consequently Δψ_m_ [309].

The detection of ROS is also an important way of assessing mitochondrial damage and function. To achieve this, usually redox-sensitive fluorophores, fluorescent proteins, and enzymatic assays are used. Some of the most relevant fluorophores are hydroethidine, dihydrorhodamine 123, and derivatives of dichlorofluorescein (DCF) that can be oxidized in the presence of ROS and detected by flow cytometry, microplate readers, or live-cell imaging [256,310]. The fluorescent reporter proteins that are redox-sensitive and can monitor ROS production are probes based on green fluorescent protein or yellow fluorescent protein and can make use of glutaredoxin-1 or peroxiredoxin to specifically determine the ROS species [260,311,312]. One of the spectrophotometric enzymatic assays that serves to monitor ROS is TCA cycle enzyme aconitase, which can be inhibited by H_2_O_2_, O_2_^−^, and peroxinitrite, and for which reduced activity is, therefore, an indicator of increased ROS production [261].

The monitoring of mitochondrial pH, ATP, and Ca^2+^ can also provide useful information on the function of mitochondria. Usually, genetically encoded and Förster resonance energy transfer (FRET)-based fluorescent protein reporters are used for this purpose. One of the reporters used to detect changes in ATP concentration is the ATeam FRET-based reporter, as well as its analogs [313]. The PercevalHR fluorescent reporter is used to detect variations in ATP/ADP ratio, which is a better indicator of energy status than ATP alone [267]. The use of bioluminescence energy transfer (BRET) probes, such as BTeam, is also able to monitor ATP fluctuations, especially when fluorescence-based methods are not suitable [266]. Finally, luciferase-based bioluminescence assays, HPLC, and ^31^P nuclear magnetic resonance (NMR) are also reliable methods for ATP measurements [263]. There are various fluorescent probes to measure free mitochondrial Ca^2+^, such as Rhod-2 acetoxymethyl, FRET-based Ca^2+^ reporter cameleons containing Ca^2+^-binding calmodulin or troponin C, circularly permuted yellow fluorescent protein-based Pericam, as well as bioluminescence-based aequorin [273,275,314,315]. Energy metabolism alterations are reflected in changes in mitochondrial pH and its measurement can provide further indications of mitochondrial function and can be used to correct experimental measurements relying on pH-sensitive fluorescent reporters. Mitochondrial pH is also measured via genetically encoded fluorescent reporters, especially those based on yellow fluorescent protein, such as mitoSypHer, but others, including pHRed and pHTomato, are also used [316,317,318,319].

Another useful indicator of mitochondrial function is the assessment of mtDNA copy number, since the mtDNA content reflects mitochondrial energy metabolism. The method employed most widely to quantify mtDNA is quantitative real-time polymerase chain reaction (PCR), although more advanced methods, such as next-generation sequencing, microarrays, and droplet digital PCR, can be used as well [280,320,321].

Moreover, the size, number, and morphology of mitochondria, which can change due to processes of fusion/fission and mitophagy, and in the presence of damaging stimuli, also represent valid indicators of mitochondrial health status. These parameters can be assessed by using labeling probes and MitoTracker^TM^ dyes, as well as immunofluorescence staining techniques and electron microscopy, which help to visualize mitochondria and examine any structural changes [283,284].

Furthermore, there are also tetrazolium salt and resazurin (7-hydroxy-3-oxo-3H-phenoxazine 10-oxide) reduction colorimetric/spectrophotometric assays that are based on the mitochondrial activity and are frequently used to evaluate cell viability but are not a reliable indicator of mitochondrial function, *per se*. The [3-(4,5-dimethylthiazol-2-yl)-2,5-diphenyltetrazolium bromide] (MTT) assay foresees the reduction in the yellow tetrazolium salt MTT into purple formazan crystals by mitochondrial succinate dehydrogenase. However, the extra-mitochondrial enzymes and other reducing agents are also capable of reducing MTT [285]. The resazurin reduction assay is also based on the reduction capacity of mitochondrial and cytosolic reductases to reduce the non-fluorescent resazurin to fluorescent pink resorufin [322]. Similar to MTT, the resazurin assays are a good indicator of cell viability, but not specific for mitochondrial activity evaluation [288].

The measurement of plasma levels and the activity of metabolites and enzymes can give significant insight into drug-induced hepatic mitochondrial dysfunction. The activity of mitochondrial enzymes, glutamate dehydrogenase, and ornithine carbamoyltransferase is a good indicator of mitochondrial structural damage and cell membrane disruption [323]. Increased plasma levels of lactate and pyruvate, on the other hand, are suggestive of reduced pyruvate oxidation [6]. Moreover, plasma levels of β-hydroxybutyrate and acetoacetate and their ratio reflect the hepatic mitochondria ratio of NADH/NAD^+^, thus indicating hepatic ETC activity [6,324]. Elevated plasma and urine levels of acyl-carnitine and acyl-glycine derivatives can indicate an alteration or inhibition of mitochondrial β-oxidation in the liver [325].

Furthermore, liver histology can also provide important information about DILI and mitochondrial dysfunction. Thus, the use of hematoxylin-eosin, or Oil red O staining, can provide evidence of the presence of lipid droplets and the type of steatosis, whereby the presence of microvesicular steatosis is a strong indication of the inhibition of fatty acid oxidation [6,326]. However, microvesicular steatosis is rarely present in pure form, and it is often combined with macrovesicular steatosis [6]. In addition to steatosis, other histopathological features, such as lobular inflammation and hepatocellular ballooning, can confirm the presence of steatohepatitis, which is also linked to mitochondrial dysfunction under various circumstances [327,328]. On the other hand, the presence of apoptosis and necrosis in the liver, despite being a valid sign, does not necessarily suggest drug-induced mitochondrial dysfunction, as these can be due to other mechanisms, as well [329]. Alterations and mitochondrial ultrastructural changes, such as the swelling and disruption of cristae, detectable by electron microscopy, offer additional information on mitochondrial dysfunction [6]. However, it is important to highlight that liver histopathology analysis is not sufficient by itself to determine drug-induced mitochondrial toxicity and should therefore be complemented by additional assays.

## 7. Concluding Remarks and Future Perspectives

There are many drugs capable of inducing liver toxicity, specifically by causing mitochondrial dysfunction. On many occasions, such effects of drugs are discovered too late in the drug development process, often during pre-clinical assessment and clinical trials, thus entailing huge costs and safety issues [1]. In fact, mitochondrial dysfunction-associated DILI has caused the interruption of clinical trials and numerous drug withdrawals from the market because it was not predicted in animal models, with many drugs even receiving black box warnings from the US Food and Drug Administration (FDA) [6,330].

For this reason, it is of critical importance to develop and use novel and relevant in vitro models and high-throughput platforms, which can help to screen many lead compounds and determine eventual mitochondrial toxicity early on during drug development, thereby reducing costs and selecting safer molecules for pre-clinical evaluation and clinical trials [1,331,332,333,334]. Furthermore, it can be beneficial to screen and study mechanisms of drugs already approved and in use. In this regard, novel 3D liver cell culture models, such as co-culture spheroids and liver organoids, could have better predictive value when assessing the liver toxicity of drugs, as they better mimic liver physiology compared to standard 2D cell cultures [331,332,335,336,337,338,339]. Another promising in vitro platform currently being developed is the liver-on-a-chip, a bioengineering and microfluidics-based system [331,340]. However, despite being more physiologically relevant, such models and platforms still require further optimization and the extensive evaluation of the sensitivity and specificity regarding hepatotoxicity [331,341].

Taking into consideration the recently demonstrated role of dysbiosis in multiple liver diseases, including DILI, there is an urgent need for novel liver in vitro models that would incorporate gut microbiota as well, therefore increasing the relevance and translational power [342,343,344]. Advanced organ-on-a-chip and 3D models replicating the gut-liver axis with added microbiota components could represent the right direction for future interdisciplinary research in the DILI field [345,346,347,348]. Moreover, the implementation of in silico prediction models, machine learning methods, and the development of comprehensive databases could further assist in selecting better and safer candidates for drug development, and predicting, with high accuracy, potential DILI [349,350,351,352].

Another important aspect to consider is the implementation of adverse outcome pathways (AOPs) that link molecular initiating events via a series of measurable key events to adverse outcomes at a biological level [353,354,355]. AOPs thereby represent a major tool, useful not only to assess toxicological features, such as liver cholestasis, steatosis, and fibrosis, but also to develop novel in vitro tests and batteries of human-based assays to study specific key events while retaining relevant translational value [354,356,357,358,359,360]. Such an approach would greatly assist in identifying appropriate assays that, together with adequate models, will be extremely advantageous for DILI prediction. Consequently, it is worth mentioning that there are different factors that can obfuscate mitochondrial toxicity and should be considered during in vitro assessment, such as the protein-binding capacity of drugs, or the expression levels of drug-metabolizing enzymes and altered bioenergetic phenotype (glycolysis-mediated ATP production) observed in several hepatoma cell lines [6].

When dealing with the evaluation of hepatic mitochondrial toxicity, it could be beneficial to take into consideration contributing factors that can affect genetic predisposition or the presence of metabolic syndrome and NAFLD. Given the increased prevalence of such disorders and other predisposing conditions, it has become clear that appropriate and advanced systems incorporating multiple cell types, cells from such patients, or genetically modified cells resembling specific predisposing phenotypes should be used more frequently in future investigations for the proper and timely assessment of hepatic mitochondrial toxicity and related liver injury [1,331]. In addition, future studies should also focus on idiosyncratic DILI. Even though the pathogenesis of this condition is not yet completely understood, there are indications that inter-individual differences, such as those related to metabolic phenotype or immune system, play an important role [361,362,363]. Novel tools that would take into account this additional layer of complexity might be capable of predicting idiosyncratic DILI and help elucidate underlying mechanisms and susceptible phenotypes. Moreover, herb-induced liver injury, due to the excessive and prolonged consumption of herbal supplements and natural products, represent another emerging problem that often has an idiosyncratic character, and therefore should also be a part of the efforts undertaken to improve liver toxicity testing [364,365].

Overall, the current advances in the field of DILI, including hepatic mitochondrial toxicity, will depend on and should rely on novel approaches combining in silico modeling and state-of-the-art human-based models to study and predict hepatotoxic events. This will undoubtedly alleviate ethical concerns related to animal use as well as the financial aspects of drug development, but will certainly require an intersectoral engagement and efforts from academia, industry, and regulatory bodies from the earliest stages, such as those showcased in the Innovative Medicines Initiative (IMI) [366,367].

## Figures and Tables

**Table 1:** DILI drugs reported to induce MPTP opening. (DILI—drug-induced liver injury; MPTP—mitochondrial permeability transition pore; JNK—c-Jun N terminal protein kinase; NSAID—nonsteroidal anti-inflammatory drug).

Drug	Therapeutic Class	Mechanism Leading to MPTP Opening	References
Acetaminophen	Analgesic	JNK activation, intracellular Fe^2+^ increase, translocation into mitochondria	[76,77,78]
Alpidem	Anxiolytic	Ligand	[73]
Amiodarone	Antiarrhythmic	Oxidative stress	[79]
Diclofenac	NSAID	Oxidative stress, intracellular Ca^2+^ increase	[74]
Disulfiram	Aversion therapy for alcoholism	Oxidative stress	[80]
Nimesulide	NSAID	Oxidative stress, intracellular Ca^2+^ increase	[81]
Salicylic acid	NSAID	Oxidative stress, intracellular Ca^2+^ increase	[82,83]
Troglitazone	Antidiabetic	JNK activation, oxidative stress, intracellular Ca^2+^ increase	[84,85,86]
Valproic acid	Antiepileptic	Oxidative stress	[69]

**Table 2:** DILI drugs reported to induce OXPHOS impairment. (DILI—drug-induced liver injury; OXPHOS—oxidative phosphorylation; ETC—electron transport chain; NSAID—nonsteroidal anti-inflammatory drug).

Drug	Therapeutic Class	Mechanism Leading to Impaired OXPHOS	References
Acetaminophen	Analgesic	Direct inhibition of ETC activity (inhibition of complexes I and II)	[93,94]
Alpidem	Anxiolytic	OXPHOS uncoupling, direct inhibition of ETC activity	[73]
Amiodarone	Antiarrhythmic	OXPHOS uncoupling, direct inhibition of ETC activity (inhibition of complexes I, II, and III)	[90]
Benzarone	Thrombolytic	OXPHOS uncoupling	[95]
Benzbromarone	Uricosuric	OXPHOS uncoupling	[95]
Buprenophrine	Therapy for opioid dependence	OXPHOS uncoupling, direct inhibition of ETC activity	[91]
Diclofenac	NSAID	OXPHOS uncoupling	[96]
Disulfiram	Aversion therapy for alcoholism	Direct inhibition of ETC activity	[80]
Ibuprofen	NSAID	OXPHOS uncoupling	[97]
Nilutamide	Antineoplastic	Direct inhibition of ETC activity (inhibition of complex I)	[92]
Nimesulide	NSAID	OXPHOS uncoupling	[81]
Perhexiline	Antianginal	OXPHOS uncoupling, direct inhibition of ETC activity (inhibition of complexes I and II)	[98]
Salicylic acid	NSAID	OXPHOS uncoupling	[96]
Tacrine	Anti-dementia	OXPHOS uncoupling	[99]
Tamoxifen	Antineoplastic	OXPHOS uncoupling, direct inhibition of ETC activity (inhibition of complexes III and IV)	[100,101]
Tetracyclines	Antibiotic	Direct inhibition of ETC activity (inhibition of complexes I and IV)	[102]
Troglitazone	Antidiabetic	Direct inhibition of ETC activity (inhibition of complex II, III, IV, and V)	[103]

**Table 3:** DILI drugs reported to induce steatosis and fatty acid β-oxidation impairment. (DILI—drug-induced liver injury; ETC—electron transport chain; mtDNA—mitochondrial DNA; NSAID—nonsteroidal anti-inflammatory drug).

Drug	Therapeutic Class	Type of Steatosis Induced	Mechanism Leading to Impaired Fatty Acid Oxidation	References
Acetaminophen	Analgesic	Microvesicular	Inhibition of fatty acid oxidation enzymes and inhibition of ETC activity	[89,93,126]
Amineptine	Antidepressant	Microvesicular	Inhibition of fatty acid oxidation enzymes and sequestration of fatty acid oxidation cofactors	[127]
Amiodarone	Antiarrhythmic	Microvesicular, macrovesicular	Inhibition of fatty acid oxidation enzymes and inhibition of ETC activity	[90,116]
Buprenophrine	Therapy for opioid dependence	Microvesicular	Inhibition of ETC activity	[91]
Didanosine	Antiretroviral	Microvesicular, macrovesicular	mtDNA depletion and inhibition of mtDNA polymerase γ	[119,128]
Fialuridine	Antiviral	Microvesicular	mtDNA depletion and inhibition of mtDNA polymerase γ	[117,119]
Ibuprofen	NSAID	Microvesicular	Inhibition of fatty acid oxidation enzymes and sequestration of fatty acid oxidation cofactors	[113]
Panadiplon	Anxiolytic	Microvesicular	Sequestration of fatty acid oxidation cofactors	[129,130]
Perhexiline	Antianginal	Microvesicular, macrovesicular	Inhibition of fatty acid oxidation enzymes and inhibition of ETC activity	[98,111]
Salicylic acid	NSAID	Microvesicular	Sequestration of fatty acid oxidation cofactors	[114]
Stavudine	Antiretroviral	Microvesicular, macrovesicular	mtDNA depletion and inhibition of mtDNA polymerase γ	[128]
Tamoxifen	Antineoplastic	Macrovesicular	Inhibition of fatty acid oxidation enzymes, inhibition of ETC activity, and mtDNA depletion	[100,107,131,132]
Tetracyclines	Antibiotic	Microvesicular	Inhibition of fatty acid oxidation enzymes	[133,134,135]
Tianeptine	Antidepressant	Microvesicular	Inhibition of fatty acid oxidation enzymes	[136]
Troglitazone	Antidiabetic	Microvesicular	Inhibition of fatty acid oxidation enzymes	[137,138]
Valproic acid	Antiepileptic	Microvesicular, macrovesicular	Inhibition of fatty acid oxidation enzymes and sequestration of fatty acid oxidation cofactors	[70,71]
Zidovudine	Antiretroviral	Microvesicular	mtDNA depletion and inhibition of mtDNA polymerase γ	[119,139]

**Table 4:** Advantages and disadvantages of the most commonly used methods and assays for mitochondrial function assessment. (MPTP—mitochondrial permeability transition pore; CO_2_—carbon dioxide; BODIPY^TM^ 493/503—4,4-difluoro-1,3,5,7,8-pentamethyl-4-bora-3a,4a-diaza-s-indacene; OCR—oxygen consumption rate; Δψ_m_—mitochondrial membrane potential; ADP—adenosine diphosphate; ATP—adenosine triphosphate; NADPH—nicotinamide adenine dinucleotide phosphate; NADH—nicotinamide adenine dinucleotide; MDR—multidrug resistance; ROS—reactive oxygen species; mtDNA—mitochondrial DNA).

Method/Assay	Advantages	Disadvantages	References
Swelling assay for MPTP opening—absorbance	Allows the multiplex assessment of mitochondrial Ca^2+^ uptake and mitochondrial swelling due to loss of the inner mitochondrial membrane integrity	Only possible in isolated mitochondria; mitochondria isolation procedure can affect shape and morphology of mitochondria, reducing reliability of obtained data	[213,214,215,216]
Swelling assay for MPTP opening—microscopy	Intact cells and fixed cells/tissue samples can be used	Low resolution; diffraction limits; artifacts due to sample preparation and fixation; difficult to estimate the actual volume of mitochondria	[216,217,218]
Fatty acid oxidation—^14^C labeled palmitate	Direct measurement of mitochondrial fatty acid oxidation efficiency	Low ^14^CO_2_ recovery rate; large inter-assay variability; use of radiolabeled compounds	[219,220]
Steatosis—staining procedures (Oil Red O, Sudan Black B, Nile Red, BODIPY^TM^ 493/503)	Simple and reproducible; allows determination of cellular localization and distribution of lipid droplets; compatible with other assays; compatible with various detection methods (microscopy, flow cytometry, plate readers)	Lower specificity; stability or the background of the signal	[221,222,223,224,225,226,227]
Steatosis—absolute lipid quantification	Specificity and sensibility; commercially available kits	Laborious procedure; does not provide information about cellular localization of lipids	[228,229,230]
OCR—Clark electrode	Simple; inexpensive	Potential artifacts due to oxygen consumption by the electrode; required cell detachment by trypsinization can affect OCR	[231,232]
OCR—Seahorse XF Flux Analyzer	Simultaneous measurement of OCR and extracellular acidification rate; reduced sample volume; high throughput	Expensive; limited to non-perfused cell population measurements	[233,234,235]
OCR—Oroboros Oxygraph-2k	Simultaneous measurement of ORC and Δψ_m_ and ADP-ATP exchange rate in suspension	Labor-intensive; low throughput	[233,236]
Mitochondrial NADPH and NADH—autofluorescence	Non-invasive; informative	Excessive exposure highly phototoxic; Exposure optimization required to improve signal-to-noise levels	[237,238,239]
Mitochondrial NADPH and NADH—fluorescent reporters	Improved sensitivity; low phototoxicity	pH sensitivity; transfection efficiency	[240,241,242,243]
Mitochondrial membrane potential variation—fluorescent dyes	Reliable and informative; compatible with various detection methods (microscopy, flow cytometry, plate readers)	Most probes are substrates of MDR transporters and mitochondrial loading can be affected; need for pharmacological inhibitors such as cyclosporin A; phototoxicity and photobleaching in confocal microscopy; possible binding to mitochondrial membrane and affecting mitochondrial respiration; some probes present high toxicity; low sensitivity	[244,245,246,247,248]
Respiratory chain complexes activity	Very informative when combined with other measurements such as OCR; useful for detecting molecular origin of mitochondrial defects	Not necessarily reflecting mitochondrial dysfunction (presence of compensatory mechanisms)	[232,249,250,251]
Mitochondrial ROS—redox-sensitive fluorophores	Relatively easy to perform and measure; compatible with live microscopy, flow cytometry, and plate readers	Not reliably attributable to mitochondrial ROS; requires some form of correction; optimization required to avoid dilution and saturation of the signal; non-linear fluorescence response; photosensitivity and pH sensitivity; auto-oxidation	[252,253,254,255,256,257]
Mitochondrial ROS—redox-sensitive fluorescent proteins	Suitable for monitoring ROS production over longer times	Lack of specificity; dye-specific pH sensitivity	[258,259,260]
Mitochondrial ROS—redox-sensitive enzymatic assays	Allows determination of enzymatic ROS origin	Provides only fixed time-point readouts	[261,262]
Mitochondrial ATP	Possible to multiplex with other fluorescent probes; reproducible; signal stability	Potential phototoxicity; potential pH and temperature sensitivity; weak signal in bioluminescence assays; expensive	[263,264,265,266,267,268]
Mitochondrial Ca^2+^	Allows specific mitochondrial targeting with genetically encoded fluorescent reporters; can be combined with Δψ_m_ measurement	Incomplete intramitochondrial accumulation of traditional fluorescent probes; possibly can cause alterations of Ca^2+^ dynamics; mostly free and not total Ca^2+^ is measured	[269,270,271,272,273,274,275,276]
Mitochondrial pH	Allows reliable calculation of the proton-motive force; good indicator of energy metabolism fluctuations	Many pH sensor probes are not specific for mitochondria; requires the use of additional mitochondrial markers	[246,277,278]
mtDNA copy number	Easy to measure and accessible	High variability in experimental procedures related to DNA extraction, quality, cross-contaminations, accuracy	[279,280,281,282]
Microscopy methods for mitochondrial morphology, size, and number	Provide more detail and insight when combined with other methods of mitochondrial dysfunction	Some probes not compatible with paraformaldehyde fixation; low transfection efficiency for targeted reporter proteins; laborious optimization of experimental protocols	[283,284]
Tetrazolium salt assay	Easy to perform; reproducible; low cost	Not reliable for mitochondrial activity assessment; endpoint assay; dependent on cell type and cell culturing	[285,286,287]
Resazurin reduction assay	Easy to perform; compatible with other assays; high sensitivity	Not reliable for mitochondrial activity assessment; requires optimization of incubation times, possibly causing cellular alterations	[288,289,290,291]

## Data Availability

Not applicable.

## Abbreviations

Δψ_m_	mitochondrial membrane potential
ADP	adenosine diphosphate
ALP	alkaline phosphatase
ALT (ALAT)	alanine aminotransferase
AOP	adverse outcome pathway
APAF1	apoptotic peptidase activating factor 1
AST (ASAT)	aspartate aminotransferase
ATP	adenosine triphosphate
Bcl-2	B cell lymphoma 2
BODIPY^TM^ 493/503	4,4-Difluoro-1,3,5,7,8-Pentamethyl-4-Bora-3a,4a-Diaza-s-Indacene
BRET	bioluminescence energy transfer
ChREBP	carbohydrate-response element binding protein
CO_2_	carbon dioxide
CoA	coenzyme A
CPT1	carnitine palmitoyltransferase 1
CYP	cytochrome P450
DAMP	danger-associated molecular pattern
DCF	dichlorofluorescein
DILI	drug-induced liver injury
DiOC6(3)	3,3′-dihexyloxacarbocyanine iodide
ETC	electron transport chain
FADH2	flavin adenine dinucleotide
FDA	Food and Drug Administration
FRET	Förster resonance energy transfer
GGT	gamma-glutamyl transpeptidase
GPx	glutathione peroxidase
GSH	glutathione
GSTM1	Glutathione S-Transferase Mu 1
GSTT1	Glutathione S-Transferase Theta 1
H_2_O_2_	hydrogen peroxide
H_2_S	hydrogen disulfide
HIF-1	hypoxia-inducible factor-1
HO·	hydroxyl radical
HPLC	high-performance liquid chromatography
IMI	Innovative Medicines Initiative
JC-1	1,1′,3,3′-tetraethyl-5,5′,6,6′-tetrachloroimidacarbocyanine iodide
JNK	c-Jun N terminal protein kinase
LC-MS	liquid chromatography-mass spectrometry
LDH	lactate dehydrogenase
MDR	multidrug resistance
MnSOD	manganese superoxide dismutase
MOMP	mitochondrial outer membrane polarization
MPTP	mitochondrial permeability transition pore
mtDNA	mitochondrial DNA
MTHFD1/2/1L	methylenetetrahydrofolate dehydrogenase 1/2/1L
MTT	3-(4,5-dimethylthiazol-2-yl)-2,5-diphenyltetrazolium bromide
NADH, NAD^+^	nicotinamide adenine dinucleotide
NADPH	nicotinamide adenine dinucleotide phosphate
NAFLD	non-alcoholic fatty liver disease
NMR	nuclear magnetic resonance
NRTIs	nucleotide reverse transcriptase inhibitors
NSAID	nonsteroidal anti-inflammatory drug
O_2_^−^	superoxide anion
OCR	oxygen consumption rate
OXPHOS	oxidative phosphorylation
PBR	peripheral benzodiazepine receptor
PCR	polymerase chain reaction
PPARα	proliferator-activated receptor alpha
Q	ubiquinone
Rh123	rhodamine-123
ROS	reactive oxygen species
SREBP-1c	sterol regulatory element-binding protein 1c
TCA	tricarboxylic acid
TMRE	tetramethyl rhodamine ethyl ester
TMRM	tetramethyl rhodamine methyl ester
TNF-α	tumor necrosis factor α
TNFR1	tumor necrosis factor receptor 1
TPP	tetraphenylphosphonium
TRAIL	tumor necrosis factor-related apoptosis-inducing ligand
XIAP	X-linked inhibitor of apoptosis protein
